# Advancements in Hybrid Cellulose-Based Films: Innovations and Applications in 2D Nano-Delivery Systems

**DOI:** 10.3390/jfb15040093

**Published:** 2024-04-04

**Authors:** Ghazaleh Ramezani, Ion Stiharu, Theo G. M. van de Ven, Vahe Nerguizian

**Affiliations:** 1 Department of Mechanical, Industrial, and Aerospace Engineering, Concordia University, Montreal, QC H3G 1M8, Canada; ghazaleh.ramezani@mail.concordia.ca; 2 Department of Chemistry, McGill University, 801 Sherbrooke St. West, Montreal, QC H3A 0B8, Canada; theo.vandeven@mcgill.ca; 3 Department of Electrical Engineering, École de Technologie Supérieure, 1100 Notre Dame West, Montreal, QC H3C 1K3, Canada; vahe.nerguizian@etsmtl.ca

**Keywords:** hybrid cellulose-based films, 2D nano-delivery systems, nanotechnology applications, biomedical engineering, advanced synthesis techniques, environmental sustainability

## Abstract

This review paper delves into the realm of hybrid cellulose-based materials and their applications in 2D nano-delivery systems. Cellulose, recognized for its biocompatibility, versatility, and renewability, serves as the core matrix for these nanomaterials. The paper offers a comprehensive overview of the latest advancements in the creation, analysis, and application of these materials, emphasizing their significance in nanotechnology and biomedical domains. It further illuminates the integration of nanomaterials and advanced synthesis techniques that have significantly improved the mechanical, chemical, and biological properties of hybrid cellulose-based materials.

## 1. Introduction

**Figure 1:**
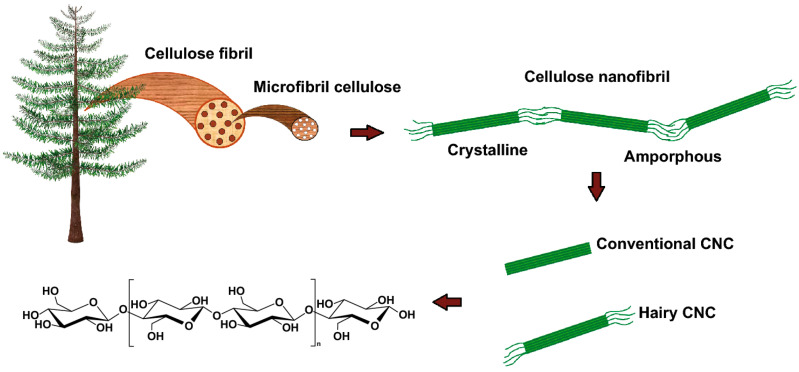
Schematic representation of cellulose structures from resources to molecular level [1]. Copyright with permission [A Review on Surface-Functionalized Cellulosic Nanostructures as Biocompatible Antibacterial Materials|Nano-Micro Letters (www.springer.com)].

In the contemporary era of sustainable material development, there is an increasing focus on products derived from cellulose, the most abundant organic compound on Earth. Cellulose’s unique combination of biocompatibility, flexibility, and sustainability has not only entrenched it as a fundamental component in traditional sectors like textiles and paper production but has also placed it at the forefront of more advanced fields. Specifically, the rise of nanotechnology and biomedical engineering has led to new advancements in cellulose-based materials, including hybrid films [1,2]. Figure 1 provides a schematic representation of cellulose structures from resources to the molecular level.

However, despite the promising potential of these materials, there are still challenges and limitations in the current research. For instance, the structure of pure cellulose has inherent shortcomings such as poor plasticity and dimensional stability and lack of antibacterial activity [3]. Moreover, the effectiveness of cellulose-based nano-delivery systems may be limited by their inability to cross the blood–brain barrier, essential for drug resistance in, e.g., epilepsy [4]. Though this review discusses the potential of hybrid cellulose-based films for applications in drug delivery, tissue engineering, and other biomedical areas, it is important to note that the materials and systems described herein are at various stages of research and development. Many of these applications are still in the experimental phase and have not yet been approved by regulatory agencies such as the Food and Drug Administration (FDA) for clinical use. The transition from laboratory research to clinical application involves extensive testing, regulatory approval, and compliance with safety and efficacy standards. Therefore, readers are cautioned that the use of these materials in medical and pharmaceutical treatments requires thorough investigation, validation, and regulatory approval before they can be considered for clinical applications. This review aims to provide an overview of current research and potential future directions in the field, rather than to suggest immediate clinical implementation.

This review explores the progress in hybrid cellulose-based films—a novel category of materials that enhance traditional cellulose products through integration with nanotechnology and chemical modifications. These breakthroughs have resulted in materials with improved mechanical properties and functionalities that can potentially be applied across various domains. Our focus lies in their crucial role in nano-delivery systems—an area experiencing significant interest for revolutionizing drug delivery, tissue engineering, and other biomedical applications [5]. 

Hybrid cellulose-based films possess distinctive properties such as controlled release of embedded active materials, targeted delivery capabilities, and biodegradability. They are anticipated to make a substantial impact on nano-delivery systems. This review presents an extensive overview of recent developments, current challenges, emerging trends, and future perspectives in this arena of hybrid cellulose-based films. It provides valuable insights for researchers, industry professionals, and policymakers who are actively involved in or keenly positioned vis-à-vis sustainable nanotechnology policy. The term ‘hybrid’ is used to describe cellulose-based films that have been enhanced through the incorporation of various nanomaterials. This integration aims to leverage the unique properties of both cellulose and nanomaterials to create films with superior mechanical, electrical, thermal, and biological properties. The ‘hybrid’ aspect refers to the combination of organic cellulose with inorganic or other organic nanomaterials, such as nanoparticles, nanotubes, and graphene, resulting in composite materials that exhibit enhanced functionalities. These hybrid films are designed for specific applications in drug delivery, environmental sensing, and biomedical engineering, among others, where their controlled release, targeted delivery capabilities, and biodegradability are crucial. The use of the term ‘hybrid’ in this context emphasizes the synergistic effects achieved by merging cellulose with nanomaterials, creating a new class of materials that are distinct from their individual components [3,6,7]. 

## 2. Synthesis Methods for Cellulose-Based Hybrid Films

Advanced synthesis techniques like electrospinning, chemical vapor deposition, and sol-gel processes have produced hybrid cellulose-based films with improved properties. 

Electrospinning is a versatile technique that can produce nanofibers from various materials, such as polymers and composites. In one study, co-electrospinning was utilized to create a composite membrane of cellulose acetate/thermoplastic polyurethanes [8,9]. This process involved optimizing parameters, including the materials’ weight percentages, the solvents’ volume ratio, and the applied voltage. The resulting composite membrane demonstrated enhanced properties, rendering it suitable for applications such as photodynamic antibacterial usage [10].

Chemical vapor deposition is another method for synthesizing hybrid films. Researchers synthesized an ultrathin, uniform organic–inorganic hybrid dielectric film using initiated CVD in a study. This hybrid dielectric was created from tetrakis-dimethyl-amino-zirconium and 2-hydroxyethyl methacrylate—a high-k inorganic material and a soft organic material, respectively. The resulting film demonstrated a high dielectric constant, low leakage current density, and high breakdown field, indicating its suitability for advanced flexible electronic applications [11,12]. 

The sol-gel process is a method for producing solid materials from small molecules. It was used to create photocatalytic coatings on cellulose fabrics through the pad-dry-cure process, using a composition that included a bifunctional anchoring agent, a crosslinking agent, and a catalyst for epoxy group polymerization. These coatings showed improved resistance to wet treatments and enhanced photocatalytic performance [13]. 

Additionally, advanced techniques like matrix-assisted pulsed laser evaporation (MAPLE) have been employed for synthesizing biopolymer thin films. The method has successfully produced thin films of polysaccharides such as dextran doped with iron oxide nanoparticles and levan. MAPLE has proven effective in obtaining thin films of sensitive materials without inducing thermal decomposition or irreversible degradation [14].

Advanced synthesis techniques contribute to the enhanced properties of hybrid cellulose-based films, including increased mechanical strength, improved drug-loading capacity, and enhanced biodegradability. However, further research is needed to optimize these methods and expand their applications [15,16]. The term ‘hybrid’ is used to emphasize the integration of cellulose with various nanomaterials, such as nanoparticles, nanotubes, and graphene, through advanced synthesis techniques like electrospinning, chemical vapor deposition, sol-gel processes, and matrix-assisted pulsed laser evaporation (MAPLE). This integration results in the formation of hybrid cellulose-based films that exhibit enhanced properties compared to pure cellulose films. 

Table 1 offers a comprehensive overview of cellulose-based 2D materials, highlighting their foundational material characteristics, recent advancements, and spectrum of applications. It outlines the challenges faced in the development and commercialization of these materials, alongside the anticipated future directions that could propel their utilization across various industries. Additionally, the table compares cellulose-based 2D materials with other nanomaterials, underlining their distinctive benefits in terms of sustainability and application diversity. Finally, it addresses real-world applications and regulatory considerations, presenting a holistic view of the potential and constraints of cellulose-based 2D materials in advancing technological and environmental solutions.

## 3. Integration of Nanomaterials

The integration of nanomaterials into cellulose films, indeed, signifies a crucial development in the creation of hybrid structures. These hybrid structures are formed by combining cellulose with a second type of nanomaterial, such as nanoparticles, nanotubes, or graphene. This combination enhances the intrinsic properties of cellulose films and introduces new functionalities, making them suitable for a wide range of applications, including nano-delivery systems. The term ’hybrid’ in this context aptly describes the synergistic effects achieved by merging nanocellulose with other nanomaterials to enhance performance and achieve tailored functionalities [17,18].

The incorporation of various nanomaterials into cellulose films is detailed in the following sections.

### 3.1. Nanoparticles 

Silver nanoparticles have been added to cellulose/starch composite films to enhance their optical properties. This addition resulted in the formation of new hydrogen bonds and a shift in the optical bandgap energy, indicating a semi-conductor effect. Characterization of this ex situ incorporation was performed using techniques such as UV–Visible spectroscopy, Fourier-Transform Infrared spectroscopy, Scanning Electron Microscopy, and Transmission Electron Microscopy, which provided insights into the structure–property relationships [19,20]. 

### 3.2. Nanotubes 

Halloysite nanotubes have been utilized to improve the mechanical properties and thermal stability of iridescent cellulose nanocrystal films. The addition of HNTs into CNC films led to a notable enhancement in tensile strength and maximum strain while maintaining the chiral nematic structure and striking iridescence of the films. This method imitates natural hybrid composite structures, like crab shells, resulting in enhanced mechanical properties and thermal stability [21,22].

### 3.3. Graphene 

Few-layer graphene has been combined with cellulose nanofibrils to produce mechanically strong and electrically conductive nanocomposite films. The CNFs-FLG hybrid was extracted from bamboo pulp and expanded graphene using an environmentally friendly process. The resulting polyethylene oxide/CNFs-FLG nanocomposite films exhibited a significant improvement in Young’s modulus, tensile strength, and electrical conductivity, making them promising candidates for future electronic devices [23,24]. 

### 3.4. Other Nanomaterials 

Manganese ferrite (MnFe_2_O_4_) nanoparticles were added to bacterial cellulose films to improve their piezoelectric properties. As a result, the nanocomposite films exhibited significantly increased piezoelectric sensitivity and are now suitable for use as low-cost, highly sensitive piezoelectric sensors [25,26].

The incorporation of these nanomaterials can potentially enhance the intrinsic properties of cellulose films and introduce new functionalities, although it is important to note that not all hybrid composite formulations will necessarily lead to favorable outcomes. The effectiveness of these enhancements depends on factors such as the type and amount of nanoparticles used, as well as the specific methods employed for their integration. [27,28]. This concludes our discussion on the integration of nanomaterials in hybrid cellulose-based films. In the following section, we will explore surface modification techniques that further enhance the properties of these films.

Table 2 provides a detailed look at how various nanomaterials, such as nanoparticles, nanotubes, and graphene, improve the properties of cellulose films. It goes further to highlight the specific applications enabled by these enhancements and the outcomes of such integration, supported by key references. This comprehensive approach demonstrates the transformative potential of nanomaterial integration in creating multifunctional cellulose films for diverse technological and biomedical applications. 

## 4. Surface Modification Techniques

### 4.1. Grafting of Polymers

Polymer grafting onto cellulose films can significantly change their properties. For example, starch acetate has been utilized as a microencapsulating agent in creating controlled-release microcapsules containing gliclazide [29]. These gliclazide microcapsules were fabricated using an emulsion solvent evaporation technique, with varying core coat ratios. In vitro studies on drug release indicated sustained release for up to 24 h from the microcapsules, depending on factors such as the size of the microcapsules, coat thickness, and SA concentration [30].

### 4.2. Coating with Bioactive Substances 

Coating cellulose films with bioactive substances can enhance their biocompatibility and functionality. Biocompatible polymers such as polylactic-co-glycolic acid, poly(ε-caprolactone), polylactic acid, poly(3-hydroxybutyrate-co-3-hydroxyvalerate), chitosan, and cellulose have been used in various biomedical applications [31,32]. These polymers can be processed into films using techniques such as augmentation, film processing, injection molding, blow molding techniques, controlled/implantable drug delivery devices, biological grafting, and nanotechnology. The resulting films exhibit improved biocompatibility with non-toxicity under mild processing conditions while reducing immunological reactions and minimizing side effects [33,34,35,36]. 

### 4.3. Other Chemical Modifications 

Other chemical modifications can be used to customize the properties of cellulose films. For example, chitosan nanocomposites have been applied in biomedical fields such as drug delivery, tissue regeneration, and wound healing. The biomedical effectiveness of chitin and chitosan compounds is closely related to the production process conditions. The use of chitosan nanocomposites in these applications has led to improved biocompatibility and functionality [37]. In summary, functionalization and surface modification strategies play a crucial role in customizing the properties of hybrid cellulose films for specific applications. These strategies can significantly enhance the performance of these films in nano-delivery systems, making them suitable for various biomedical uses.

Table 3 outlines the diverse surface modification techniques applied to cellulose films, detailing the methods employed, their resultant enhancements, and the corresponding scientific references. These modifications significantly alter the physical and chemical properties of cellulose films, improving their applicability in biomedical and technological fields. The table is segmented into three primary modification strategies, each aiming to enhance the biocompatibility, functionality, and overall performance of the cellulose films in various applications: grafting of polymers, coating with bioactive substances, and other chemical modifications..

## 5. Recent Studies and Publications 

### 5.1. Customization of Cellulose Nanostructures for Specific Delivery Challenges 

Recent research has indicated the potential of cellulose nanostructures for targeted drug delivery. One-dimensional nanostructures, like nanorods, nanofibers, or nanotubes, have high drug-loading capacities and improved targeting due to their high surface-to-volume ratios. These structures can be tailored to target different cells by altering the ligands used. Moreover, they can react to changes in the target environment, enabling drug release only at the specific site [38].

### 5.2. Cellulose-Based Systems for Hydrophobic Drug Delivery

#### 5.2.1. Antibacterial Cellulose-Based Materials

**Figure 2:**
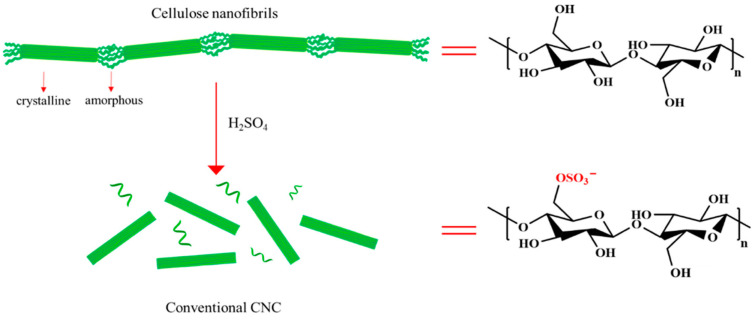
Schematic illustration of the synthesis of the conventional cellulose nanocrystalline from cellulose nanofibrils under acid hydrolysis [39]. Copyright with permission [Biotemplated Hollow Mesoporous Silica Particles as Efficient Carriers for Drug Delivery|ACS Applied Bio Materials].

Research has led to the development of antibacterial nanocrystalline cellulose through a novel functionalization process. This process involves the integration of aldehyde groups into hairy nanocrystalline cellulose, enabling the direct attachment of natural antibacterial agents such as lysozyme and nisin. The effectiveness of this method has been demonstrated through its ability to maintain the antibacterial activity of these agents over extended periods, signifying a promising approach for antibacterial applications [39]. Figure 2 presents a schematic illustration of the synthesis process of conventional nanocrystalline cellulose from cellulose nanofibrils via acid hydrolysis, highlighting its application in the development of antibacterial cellulose-based materials. This novel functionalization process involves integrating aldehyde groups into hairy nanocrystalline cellulose, facilitating the direct attachment of natural antibacterial agents such as lysozyme and nisin. The method’s effectiveness is underscored by its capability to preserve the antibacterial activity of these agents over extended periods, offering a promising avenue for enhanced antibacterial applications [39].

#### 5.2.2. Antibacterial and Biofilm-Disrupting Hydrogels 

**Figure 3:**
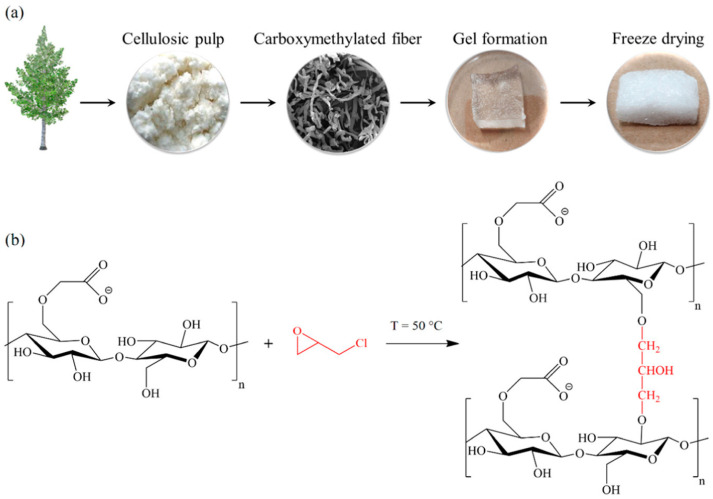
(**a**) Representative images of different stages of the gel formation. (**b**) Schematic of the reaction of carboxymethyl cellulose fiber and epichlorohydrin to form the gel [40]. Copyright with permission [Highly Absorbent Antibacterial and Biofilm-Disrupting Hydrogels from Cellulose for Wound Dressing Applications|ACS Applied Materials & Interfaces].

Innovations in cellulose for wound-dressing applications have resulted in the creation of highly absorbent antibacterial and biofilm-disrupting hydrogels. These hydrogels are produced by covalently bonding ε-poly-L-lysine to carboxyl-modified cellulosic hydrogels, exhibiting significant bactericidal efficacy and compatibility with mammalian cells. This breakthrough suggests a potent solution for wound-dressing materials (Figure 3) [40]. 

#### 5.2.3. Antibacterial Pickering Emulsions 

Further exploration into antibacterial cellulose-based materials has given rise to antibacterial Pickering emulsions stabilized by bifunctional hairy nanocellulose. These emulsions utilize cellulose nanocrystals modified with aldehyde and carboxylic acid groups, offering stability without surfactants and demonstrating enhanced antibacterial properties when combined with essential oils like carvacrol [41]. 

#### 5.2.4. Green Extraction of Antibacterial Cellulose-Based Nanofibers 

**Figure 4:**
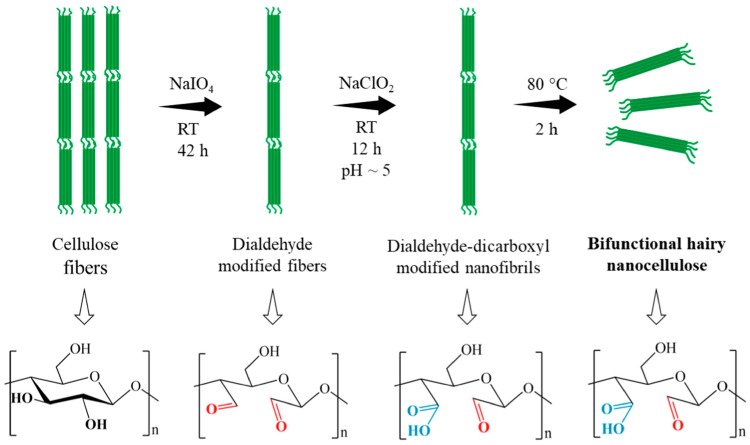
Schematic of cellulose reaction with sodium periodate to produce dialdehyde-modified cellulose (DAMC), followed by reaction with sodium chlorite to synthesize dialdehyde-dicarboxylate-modified nanofibrils, and finally BHNC isolation through heat treatment [6]. Copyright with permission [Antibacterial Pickering emulsions stabilized by bifunctional hairy nanocellulose—ScienceDirect].

A noteworthy advancement has been made in the extraction of antibacterial cellulose-based nanofibers from natural sources such as pine cones. Utilizing an eco-friendly method involving thermo-oxidation with H_2_O_2_, these extracted nanofibers have shown potent antibacterial activity against common pathogens, marking a significant step forward in green extraction technologies [42,43]. Figure 4 illustrates the chemical modification process of cellulose to enhance its antibacterial properties through a green extraction methodology. Initially, cellulose reacts with sodium periodate to produce dialdehyde-modified cellulose (DAMC). Subsequently, it undergoes a reaction with sodium chlorite to synthesize dialdehyde-dicarboxylate-modified nanofibrils. The process concludes with the isolation of bifunctional hairy nanocellulose (BHNC) through heat treatment. This schematic represents a significant advancement in the extraction of antibacterial cellulose-based nanofibers from natural sources like pine cones, using an eco-friendly thermo-oxidation method with H_2_O_2_, demonstrating potent antibacterial activity against common pathogens [6].

#### 5.2.5. Superhydrophobic and Antibacterial Cellulose-Based Fibers and Fabrics 

Recent reviews have highlighted progress in the development of superhydrophobic and antibacterial cellulose-based fibers and fabrics. These advancements focus on enhancing antibacterial properties through surface structuring, chemical modifications, and the incorporation of antibacterial agents, aiming to balance biocompatibility and biodegradability [44]. 

#### 5.2.6. Edible Carboxymethyl Cellulose Films with Natural Antibacterial Agents 

Studies have also ventured into edible carboxymethyl cellulose films incorporated with natural antibacterial agents like lysozyme. These films not only demonstrate improved mechanical and water resistance properties but also exhibit significant antimicrobial efficacy against food-borne pathogens, showcasing the potential for food-packaging applications [45].

#### 5.2.7. Cellulose-Based Hydrogels for Antibacterial Wound Dressing 

Additionally, cellulose-based hydrogels have been identified as promising materials for antibacterial wound dressing, capable of mimicking the skin’s microenvironment. The exploration of cellulose from diverse sources for hydrogel production emphasizes the importance of biocompatibility and biodegradability in creating effective wound-healing solutions [46]. 

These developments in hybrid cellulose-based films and materials underscore the versatile and significant potential of cellulose in crafting innovative and effective antibacterial solutions for a broad range of applications. 

Table 4 compiles significant research findings and developments in the realm of cellulose-based 2D materials, emphasizing their potential in drug delivery, antibacterial applications, and comparative advantages over other nanomaterials. Each row delineates a specific area of research, detailing key insights into the customization of cellulose nanostructures for enhanced drug delivery, the development of novel antibacterial materials, and the creation of innovative hydrogels and emulsions with significant medical and commercial implications. Furthermore, the table provides a comparative analysis, illustrating the unique attributes of cellulose-based materials in terms of sustainability, biodegradability, and application versatility. The referenced studies underscore the ongoing innovation and potential of cellulose-based materials in addressing contemporary challenges in healthcare, environmental sustainability, and material science.

### 5.3. Drug Delivery 

#### 5.3.1. Biotemplated Hollow Mesoporous Silica Particles 

Recent studies have introduced hollow-shaped porous silica materials, utilizing a biotemplate-directed method with cellulose nanocrystals as the template. These materials, including porous hollow silica nanorods and ultraporous sponge-like structures, have showcased their efficiency in drug delivery and dye removal applications. Notably, the ultraporous variants exhibit superior adsorption capacities, marking a significant stride in material design for therapeutic applications [47]. 

#### 5.3.2. Stimuli-Responsive Chitosan 

The versatility of chitosan has been explored through its responsiveness to both internal (pH, temperature, enzymes) and external (ultrasound, light, magnetic fields) stimuli. This adaptability positions chitosan as a formidable platform for controlled drug delivery, with current designs emphasizing the material’s efficiency in releasing bioactive agents under specific conditions [48]. 

#### 5.3.3. Hairy Nanocellulose-Based Supramolecular Architectures 

Innovations in biopolymer-based nanosponges have led to the creation of trifunctional structures by cross-linking beta-cyclodextrin ethylene diamine with bifunctional hairy nanocellulose. These complexes, characterized by their unique morphological and structural properties, have demonstrated potential in capturing and sustaining the release of drug molecules like doxorubicin, with notable pH responsiveness [49]. 

#### 5.3.4. Internally Bridged Nanosilica 

Research has yielded monodisperse colloids with a distinctive sea urchin-like structure, internally bridged for hosting drugs within their framework. These pH-responsive nanocomposites have been tested for their biocompatibility as nanocarriers, presenting a novel approach to controllable drug delivery mechanisms [50]. 

#### 5.3.5. Hollow Silica-Based Materials 

Molecular dynamics simulations have been employed to investigate the transfer processes of drugs using hollow silica particles, focusing on the optimization of surface wettability, pore size, and flow velocity. This analytical approach provides critical insights into enhancing drug delivery efficiency through structural optimization [51]. 

#### 5.3.6. Chitosan/Hyaluronic Acid Nanoparticles 

The development of oxidative stress and pH-responsive nanoparticles from chitosan/hyaluronic acid has been reported. These nanoparticles excel in the encapsulation and controlled release of small molecules and proteins, triggered by specific endogenous conditions. Their high encapsulation efficiency underscores the potential for targeted therapeutic applications [52]. 

#### 5.3.7. Reusable Green Adsorbent Aerogel for Dye Removal 

A study explores the development of a reusable green adsorbent aerogel. This aerogel is made from crosslinked hairy nanocrystalline cellulose and modified chitosan, showcasing its effectiveness in dye removal. The research highlights the potential of this eco-friendly material in environmental applications, particularly in water purification [53]. 

#### 5.3.8. Stimuli-Responsive Chitosan for Efficient Delivery of Bioactive Agents 

In another study, the use of stimuli-responsive chitosan as a platform for drug delivery systems is reviewed. This material can respond to various internal and external stimuli, making it highly suitable for controlled and targeted delivery of bioactive agents. The paper discusses the design of chitosan-based architectures that can efficiently load and release drugs at desired sites, emphasizing the potential of chitosan in biomedical applications [54].

#### 5.3.9. Detection of Biomolecules

Cellulose-based systems have been effectively used for delivering hydrophobic drugs. For instance, one study showcased the use of cellulose nanocrystals derived from rubber wood as a delivery vehicle for hesperidin, a hydrophobic drug. The CNCs were modified and coated with a cationic surfactant to create a drug delivery system that exhibited promising drug release performance. Another study introduced a solid self-nanoemulsifying drug delivery system using innovative cellulose-based microparticles as adsorptive carriers for the lipophilic drug celecoxib. This system combined the benefits of liquid-SNEDDS with those of solid dosage forms, particularly stability, while preserving the in vitro release performance of the liquid formulation [55,56,57]. Additionally, the exploration of theoretical models for optimizing antibody–antigen interactions opens new avenues in biomolecule detection, leveraging computational techniques to enhance the selectivity and functionality of antibody-based filtration systems [58].

#### 5.3.10. Cellulose-Based Systems in Targeted Cancer Therapy 

Cellulose-based systems have been explored for targeted cancer therapy. For instance, one-dimensional nanostructures have been used to encase anticancer drugs into cellulose nanofibers, providing a novel approach for drug delivery systems [59,60]. Also, DNA nanomaterials with advanced molecular recognition capabilities and biocompatibility have acted as carriers for the CRISPR/Cas gene-editing system in cancer therapy [55].

In conclusion, recent research has highlighted the potential of cellulose-based systems in drug delivery, particularly for hydrophobic drugs and targeted cancer therapy. These versatile systems tackle specific delivery challenges and present exciting prospects for future advancements in drug delivery research and development [61]. 

## 6. Emerging Trends and Challenges 

In recent years, there has been a significant trend in the development of stimuli-responsive cellulose-based materials. These materials are designed to react to specific stimuli such as temperature, light, electrical signals, magnetic fields, and humidity. The choice of cellulose as a base material is favored because of its sustainability and renewability [62,63]. 

Cellulose-based materials that respond to stimuli have been used in a wide range of applications, including drug delivery. Bacterial cellulose fibers, for example, have shown promise as a sustainable platform for drug delivery. By designing these materials to react to specific stimuli within the body, they can enable targeted and controlled release of drugs. This approach has the potential to enhance the effectiveness of drug therapies and minimize the side effects [64]. 

Additionally, the exploration of theoretical models for optimizing antibody–antigen interactions opens new avenues in biomolecule detection, leveraging computational techniques to enhance the selectivity and functionality of antibody-based filtration systems [65].

However, the development and use of these materials pose several challenges. Scalability is one of the main challenges. Although they exhibit promise in laboratory settings, scaling up production to industrial levels can be difficult due to factors such as cost, complexity of production processes, and quality control [66].

Another challenge is compatibility with a broader range of pharmaceutical compounds. Although cellulose-based materials have been successfully used with specific drugs, further exploration of their compatibility with other pharmaceutical compounds is necessary. This is especially important for the delivery of protein-based therapeutics, which often requires specific conditions for stability and activity [67]. 

Furthermore, the prospective integration of these materials with advanced technologies such as the Internet of Things (IoT) has yet to emerge as a present concern but might become critical in the future, contingent on the security of internet traffic. At this juncture, the full potential of the IoT in healthcare, particularly in drug delivery systems, remains largely untapped due to these security considerations. Without stringent security measures in place, IoT-enabled drug delivery could pose significant risks, likening it to a precarious situation akin to handling a loaded gun. Thus, the advancement of the IoT in drug delivery hinges on overcoming these security challenges to ensure it serves as a beneficial tool rather than a potential threat [68,69,70]. 

In conclusion, though stimuli-responsive cellulose-based materials exhibit considerable potential for targeted drug delivery and other applications, addressing the existing significant challenges remain imperative. To fully realize the vision of personalized medicine—creating specific formulations tailored to individual patients—further research and development are essential. This endeavor will not only require advancements in materials science and pharmaceutical sciences but also significant contributions from information technology and artificial intelligence. Collaboration across these diverse fields is crucial to harnessing the capabilities of cellulose-based materials for personalized therapeutic solutions, illustrating a comprehensive approach to overcoming the hurdles that currently impede progress [71,72,73].

Table 5 synthesizes cutting-edge research on cellulose-based materials, highlighting their key achievements, methodologies, and potential applications across various fields. It illustrates the versatility of cellulose nanostructures, ranging from enhanced drug delivery systems to antibacterial and biofilm-disrupting materials. The table emphasizes the innovative approaches in customizing cellulose nanostructures for targeted applications, developing novel antibacterial materials, and creating sustainable solutions for environmental and medical challenges. Each entry details the focus of the study, the unique achievements made, the methodology applied to achieve these outcomes, and the potential real-world applications, supported by references to the original research. This overview showcases the significant potential of cellulose-based materials in contributing to advancements in healthcare, environmental sustainability, and material science.

## 7. Comparative Analysis of Cellulose-based 2D Materials and Other Nanomaterials 

Cellulose-based 2D materials have been attracting significant attention due to their unique properties and potential applications in various fields. Derived from cellulose, one of the most abundant renewable materials, they are known for their mechanical robustness, biocompatibility, and biodegradability. In comparison to other nanomaterials, cellulose-based 2D materials offer several advantages. They are low-cost, abundant, and environmentally friendly due to their biodegradability. Moreover, they exhibit a wide variety of fibers that can be manipulated at the nano-, micro-, and macroscales to produce synthetic cellulose-based active materials [74]. 

Cellulose-based 2D materials find various applications. For instance, they are utilized as potential CO_2_ adsorbents due to their high adsorption capacity. They have also been combined with MXenes, a group of 2D metal carbides and nitrides, to create composite electrodes for supercapacitors [75]. 

Graphene-based 2D nanomaterials have been extensively researched for their potential applications in various sectors due to their large surface area and anisotropic physicochemical properties, making them suitable for biomedical and agroecological applications. However, cellulose-based 2D materials have distinctive properties that distinguish them. For instance, they can be assembled into rod-like crystallites in either 2D or 3D forms, and modifications within the oligosaccharide core can impact molecular packing, resulting in the formation of unique structures [76,77]. 

Cellulose nanofibrils/nanofibers are a type of cellulose-based 2D material widely used in environmental science applications. They are favored for their one-dimensional nanostructure, high specific surface area, excellent biodegradability, low cost, and sustainability. In conclusion, though both cellulose-based 2D materials and other nanomaterials like graphene have their unique properties and applications, their environmental friendliness, versatility, and ability to be manipulated at different scales make cellulose-based 2D materials stand out for various applications [78]. 

### 7.1. Detailed Comparative Analysis

- Electrical Conductivity: Graphene is well known for its remarkable electrical conductivity, making it the material of choice for electronic applications. In contrast, cellulose-based 2D materials generally do not exhibit high electrical conductivity but can be modified for specific electronic applications where biodegradability is a priority [79].
- Thermal Stability: Carbon nanotubes, particularly multi-walled carbon nanotubes, are renowned for their exceptional thermal stability. This quality makes them ideal for use in applications that involve exposure to high temperatures. Though cellulose-based materials may not rival the thermal stability of CNTs, they do possess thermal properties suitable for applications such as packaging and insulation [80].
- Biodegradability: Cellulose-based 2D materials outperform graphene and CNTs in terms of biodegradability, making them more appropriate for environmentally friendly applications [81].

Table 6 provides a comparative overview of cellulose-based 2D materials versus other prominent nanomaterials like graphene and carbon nanotubes (CNTs), highlighting key properties such as electrical conductivity, thermal stability, and biodegradability. It emphasizes the unique advantages of cellulose-based materials, including their environmental friendliness, versatility, and mechanical robustness, alongside their applications and modifications at various scales. The analysis aims to underline the distinctive features and potential applications of cellulose-based 2D materials in contrast to their nanomaterial counterparts, showcasing their suitability for a broad spectrum of applications, from environmental sciences to electronics and biomedicine.

### 7.2. Application Suitability

- Biomedical Applications: Cellulose-based 2D materials are better suited for biomedical applications, like drug delivery and tissue engineering, because of their biocompatibility and biodegradability. Graphene and its derivatives, although valuable in medicine, may raise concerns regarding biodegradability and long-term effects on the body [82].
- Environmental Sensing: MXenes are a group of 2D metal carbides and nitrides that show potential in gas-sensing applications due to their high electrical conductivity and functional groups. On the other hand, materials based on cellulose may not provide comparable conductivity but can be used in environmental applications where recyclability is a key consideration [83,84].
- Electronic Devices: Graphene’s superior electrical properties make it more suitable for high-performance electronic devices. However, in applications where environmental impact is a concern or for disposable electronics, cellulose-based materials could be preferred [72,75].

### 7.3. Unique Properties

- Renewable Sourcing: Cellulose-based 2D materials are derived from renewable resources, providing a significant advantage over other nanomaterials that may require more energy-intensive production processes or use non-renewable resources [85].
- Sustainable Production: The production of 2D materials based on cellulose can be more sustainable, with lower CO_2_ footprints compared to producing other nanomaterials such as graphene from plastic waste [86].

### 7.4. Limitations and Challenges 

While hybrid cellulose-based films offer numerous advantages, there are several limitations and challenges that need to be addressed:

- Biodegradability: Although cellulose is biodegradable, the incorporation of certain nanomaterials into hybrid films may affect their overall biodegradability. For instance, some hybrid films may not be fully biodegradable due to the presence of non-biodegradable nanomaterials, despite the biodegradability of the cellulose component. However, the decomposition of the cellulose would release the solid non-biodegradable components that could be easily collected for recycling purposes.
- Scalability: The production of hybrid cellulose-based films on an industrial scale may face challenges related to scalability and cost effectiveness. The synthesis and integration of nanomaterials into cellulose matrices can be complex and may require sophisticated techniques, which can increase production costs.
- Performance Consistency: Achieving consistent performance across different batches of hybrid films can be challenging. Variations in the synthesis process or the quality of raw materials can lead to inconsistencies in the properties of the final films.
- Environmental Impact: Though cellulose-based films are generally considered environmentally friendly, the environmental impact of the nanomaterials used in hybrid films needs to be carefully assessed. The potential release of nanoparticles into the environment and their interactions with biological systems are areas of concern that require further research.
- Regulatory Hurdles: The use of hybrid cellulose-based films in certain applications, such as food packaging or biomedical devices, may be subject to regulatory hurdles. Ensuring compliance with safety and health regulations is crucial for the commercialization of these materials.

### 7.5. Future Directions and Opportunities

In the next section, we will discuss recent advances in the field that aim to address some of these challenges and enhance the performance of hybrid cellulose-based films.

- Scalability: Scaling up the production of cellulose-based 2D materials while maintaining their quality can be more challenging compared to the established production processes for graphene and CNTs [87].
- Purity and Functionalization: Achieving high purity and functionalization of cellulose-based 2D materials can be more challenging compared to other nanomaterials, which may limit their application in certain high-tech areas [88].

Recent Advances

- Graphene Manufacturing: Recent advancements in manufacturing graphene from plastic waste have the potential to reduce the environmental impact of graphene production [89].
- Hybrid Composites: The development of hybrid composites demonstrates the potential for combining cellulose properties with other nanomaterials, such as nitrogen-doped MWCNTs, grafted with carboxymethyl cellulose, to enhance their applications [90,91].

By incorporating detailed comparisons and discussions, the section will provide a clearer understanding of the position of cellulose-based 2D materials in relation to other nanomaterials. This will highlight their unique advantages and potential applications, while also acknowledging the challenges they face. 

## 8. Case Studies and Potential Applications

Cellulose-based 2D nano-delivery systems have been explored in various fields such as pharmaceuticals, environmental applications, and biomedical engineering. Recent case studies demonstrate their practical applications.

- Pharmaceutical Industry

Smart Acetaminophen Delivery: Core-shell nanoparticles were developed for smart drug delivery of acetaminophen using cellulose acetate and polyvinylpyrrolidone. These nanoparticles exhibited a biphasic release, offering rapid therapeutic action followed by sustained release over 30 h. This feature is beneficial for maintaining an effective blood drug concentration [56,92].

- Environmental Applications

Eucalyptus Essential Oil Delivery: Nanofibrillated and microfibrillated cellulose-based materials were utilized to form 3D networks for encapsulating and delivering eucalyptus essential oil. These systems were tailored for dermic and respiratory applications, displaying precise and consistent release kinetics essential for long-lasting therapeutic effects [93,94].

- Biomedical Engineering

Electrospun Nanofiber Scaffolds: Advances in electrospinning have led to the development of ultrafine nanofiber scaffolds from cellulose derivatives. These scaffolds are used in biomedical applications including transdermal systems, antibacterial agents, wound dressing, cancer treatment, and as carriers for growth factors and stem cell delivery systems [88,95]. 

Thermosensitive Drug Delivery: A study on PNIPAM/Hexakis nanocomposites has highlighted their potential as thermosensitive drug delivery systems due to their ability to respond to temperature changes. This property is advantageous for achieving controlled drug release in biomedical and pharmaceutical applications [96,97].

- Marketed Nano-delivery Systems

Approved Therapeutic Nanoparticles: The pharmaceutical industry has witnessed the approval of a variety of therapeutic nanoparticles, including Epaxal^®^, an aluminum-free hepatitis ’A’ vaccine, and Puricase^®^, designed for tophaceous gout. These products illustrate the successful implementation of nanoparticle-based drug delivery systems in the market [98,99].

- Optimization and Quality Control

Bacterial Nanocellulose Production: Research on bacterial nanocellulose has been focused on optimizing production and quality control for its use as a non-active pharmaceutical ingredient, to develop sustainable biomaterials to replace non-renewable sources in delivery systems [100].

- Plant-Based Compound Delivery

Nano-Formulated Plant-Based Compounds: The development of nano vehicles for encapsulating plant-based compounds has been explored to combat bacterial infections. These nano-formulated compounds can disrupt cell membranes, inhibit enzyme activity, and interfere with biofilm formation. This displays the versatility of cellulose-based nano-delivery systems in pathogen treatment. These case studies illustrate the diverse applications of cellulose-based 2D nano-delivery systems across different sectors, highlighting their potential to revolutionize drug delivery and therapeutic strategies [100].

- Biodegradable Medical Implants

A study on cellulose-acetate-based composites used as coatings for biodegradable magnesium implants for trauma showed promising results. The composite coating reduced the biodegradation rate of the magnesium implants, known to promote bone healing and exhibit adequate mechanical strength during their biodegradation in the bone-healing process [101]. Another study on Mg–Ca–Zn biodegradable alloys used as orthopedic implants demonstrated high biocompatibility and excellent mechanical properties, making them suitable for small bones of the feet and hands, ankles, or small joints [102,103]. 

## 9. Environmental Impact 

Cellulose-based 2D nanomaterials have demonstrated remarkable potential in reducing energy consumption for heat transfer operations, offering a sustainable alternative. Through environmental impact analysis, it has been revealed that these materials can be utilized on a large scale in automobile industries and cooling processes [104,105]. 

The environmental impact of producing and disposing of hybrid cellulose-based films, especially in the context of 2D nano-delivery systems, can be assessed through life cycle analysis, carbon footprint evaluation, and comparison with traditional materials [106,107].

- Carbon Footprint

The carbon footprint of a product refers to the total amount of greenhouse gases, including carbon dioxide and methane, emitted into the atmosphere during its production, use, and end-of-life phases. The increased use of fossil-based plastic in food packaging has contributed to higher levels of plastic waste, carbon footprints, and global warming. This has prompted the exploration of alternatives like cellulose-based hydrogels for biodegradable food packaging [108,109].

- Comparison with Traditional Materials

Traditional packaging systems are often made of petroleum-based materials, which raise environmental concerns due to their limited biodegradability and the pollution they cause. In contrast, cellulose-based films are derived from renewable resources and are biodegradable, making them a more environmentally friendly alternative. However, these films often lack antioxidant and antimicrobial activities that are crucial for food preservation. To address this issue, researchers have developed active films by incorporating antioxidants and antimicrobial agents into the films [110]. 

Moreover, the drawbacks of petrochemical polymer-based packaging, such as extreme depletion of fossil resources, excessive carbon footprints of products, and environmental pollution from nonbiodegradable plastic packages have prompted scientists to develop novel packaging materials from nature-inspired biopolymers like cellulose. In conclusion, hybrid cellulose-based films can significantly reduce the environmental impact of production and disposal compared to traditional materials, making them a more sustainable choice [111].

- Energy Storage

MXenes, a new class of advanced 2D nanomaterials, have become prominent among various types of electrode materials for electrochemical energy storage devices. Due to their distinctive layered structures, enhanced electrical and thermal conductivity, superior charge carrier mobility, and strong mechanical properties, these materials have introduced an intriguing opportunity in functional materials. Ongoing research aims to address the challenge of aggregation and nanosheet restacking that reduces the accessibility of the active surface sites of MXene materials for electrolyte ions [112].

- Water Purification

Cellulose nanofibrils/nanofibers are extensively utilized in environmental science applications, particularly for water purification. Their high specific surface area, excellent biodegradability, low cost, and sustainability make CNFs well suited for the removal of metal ions, anions, organic dyes, oils, and bio-contents from water [113,114].

- 4D Printing

Cellulose-based materials have also been utilized in 4D printing. Printed cellulosic materials can transform from a 1D strand or 2D surface into a 3D shape in response to an external stimulus. This technology has potential applications in numerous fields, such as biomedicine, tissue engineering, wearable devices, and environmental science [115,116]. 

Here are some recent studies and publications that demonstrate the practical applications of these materials:

- Dermic and Respiratory Applications

A study was conducted on the design, development, and optimization of drug delivery systems using nanofibrillated and microfibrillated cellulose-based materials as 3D networks encapsulating eucalyptus essential oil molecules for dermal and respiratory applications. The optimized porous structures retained the desired molecules, leading to controlled and uniform release kinetics over time—a crucial aspect in developing effective drug delivery systems in biomaterials fields [117].

- Biomedical Applications of Chitosan Nanocomposite

Chitosan nanocomposites have found use in various biomedical applications including drug delivery, tissue regeneration, and wound healing. The physicochemical properties of chitin and chitosan compounds are closely tied to the conditions of their production process, influencing their biomedical activity. These materials have also been studied for their potential to substitute non-renewable fiber sources in the creation of non-woven delivery systems [118].

- Aerogels for Biomedical Applications

Cellulose nanofiber-based aerogels are widely utilized in the biomedical field because of their biocompatibility, renewability, and biodegradability. These aerogels find applications in sustainable antibiotic delivery for wound healing, preparation of scaffolds for tissue cultures, development of drug delivery systems, biosensing, and as an antimicrobial film for wound healing [56].

- Thermosensitive Drug Delivery Systems

Thermosensitive drug delivery systems, like PNIPAM/Hexakis, have been the focus of research in nanobiotechnology due to their multifunctional properties. These systems show potential for a variety of applications, including cell delivery, and have been investigated for their ability to enhance the delivery of biomolecules, genes, and drugs [119].

- Electrospun Nanofiber in Drug Delivery Systems

Electrospun nanofiber scaffolds have been developed for drug delivery systems, offering benefits such as high specificity and a porous structure suitable for the delivery of biomolecules, genes, and drugs. These nanofibers have been applied in transdermal systems, antibacterial agents, wound dressing, cancer treatment, scaffolds for growth factor delivery, and carriers for stem cell delivery systems [38].

- Biopolymeric Auto-Fluorescent Micro- and Nanogels

Multifunctional biopolymeric auto-fluorescent micro- and nanogels have been developed as a platform for biomedical applications, specifically in the field of theragnostics for advanced healthcare [38,120].

These real-world applications demonstrate the potential of hybrid cellulose-based films and related materials in addressing various challenges in drug delivery and biomedical applications. The case studies and practical insights illustrate that these materials are not only theoretically promising but are also actively being developed into products and solutions with significant potential impact on healthcare and medicine. 

Cellulose-based 2D materials possess a variety of unique properties that make them appealing for a wide range of applications, such as those detailed below.

- Biodegradability and Renewable Sourcing: Cellulose is one of the most abundant renewable materials on Earth, making it biodegradable and CO_2_-neutral in the long run. This status is conditional on sustainable practices, such as replanting trees to replace those harvested for cellulose production—ensuring CO_2_ neutrality as these new plantings mature. Cellulose is available in a wide variety of fibers globally, which contributes to the sustainability and potential environmental friendliness of cellulose-based materials [121,122].
- Mechanical Robustness: Cellulose and cellulose-based composite materials are well known for their mechanical strength, which is especially advantageous in applications like biodegradable medical implants, where the material must retain its structural integrity under physiological conditions [62,123].
- Unique Nanostructuring: Nanostructuring cellulose-based 2D materials can result in unique properties. For instance, cellulose nanofibrils/nanofibers possess a high specific surface area due to their one-dimensional nanostructure, which is advantageous for applications like water purification [124,125].
- Electrical Conductivity: MXenes are a class of 2D nanomaterials with metal-like electrical conductivity, making them promising electrode materials for energy storage devices. When combined with cellulose, these materials can overcome common issues such as low mechanical strength and restacking, which are often associated with MXenes [126,127].
- Optical Properties: Cellulose-based materials can exhibit unique optical properties. For instance, hemicellulose nanocrystals derived from industrial biowastes demonstrate excellent dispersibility in water and are suitable for use in applications involving one-dimensional (1D) carbon nanotube nano-inks as well as two-dimensional (2D) transition metal dichalcogenide nanozymes [128,129].
- Thermal Stability: Cellulose-based materials, like the ones used in MXene/cellulose-based electrodes, are recognized for their thermal stability. This characteristic is essential for applications involving high temperatures or requiring materials to maintain their properties under thermal stress [130].
- Hydrophilicity: Cellulose-based materials, like graphene oxide-cellulose nanocrystal hybrid membranes, exhibit improved surface hydrophilicity. This characteristic is advantageous for applications requiring water permeability, such as wastewater treatment [131].

Cellulose-based 2D materials possess unique properties that make them well suited for a wide range of applications, including biodegradable medical implants, energy storage, and water purification. Ongoing research in this field is expected to unveil further potential applications and benefits of these materials. 

### 9.1. Detailed Applications of Cellulose-Based 2D Materials 

Cellulose-based 2D materials have been extensively researched for their potential in various biomedical applications. They possess unique properties such as customizable surface modification, favorable optical transparency, good hydrophilicity, excellent biocompatibility, and mechanical properties that are both remarkable and especially suited for the demands of various applications, especially in the biomedical field [132,133].

- Drug Delivery

**Figure 5:**
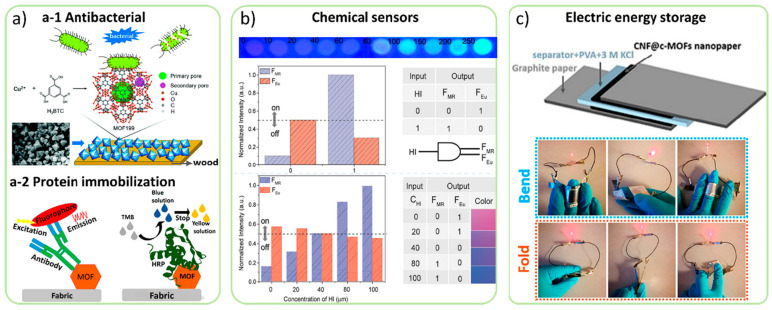
(**a**) Applications of MOF/cellulose composite as antibacterial material and for protein immobilization. (**a-1**) Schematics of the fabrication of MOF wood composite materials and their antibacterial mechanism. (**a-2**) Illustration of antibody or enzyme immobilized by MOF on fabric substrate. (**b**) MOF/cellulose hydrogel exhibited a color transition upon sensing histamine (HI) vapor and the truth table of the logic analytical device for HI monitoring. (**c**) Photograph of a CNF@c-MOF double-layer supercapacitor device and an LED powered by devices in series under different deformations. Copyright with permission [Review on design strategies and applications of metal-organic framework-cellulose composites—ScienceDirect].

Cellulose-based materials are widely used in drug delivery to create sustained-release systems. For instance, novel cellulose-based microparticles have been utilized as adsorptive carriers to develop solid self-nano-emulsifying drug delivery systems. These systems aim to improve the oral bioavailability of poorly soluble lipophilic drugs while maintaining stability and release behaviors. Moreover, cellulose and its derivatives have been employed as excipients in controlled drug delivery systems, allowing them to modify the solubility and gelling behavior of drugs for controlling release profiles [134,135,136]. Figure 5 illustrates various applications of MOF/cellulose composites, including their use as antibacterial materials and for protein immobilization. In Figure 5a-1, schematics depict the fabrication of MOF wood composite materials and their antibacterial mechanism. Figure 5a-2 shows an illustration of antibodies or enzymes immobilized by MOF on a fabric substrate. Figure 5b demonstrates how MOF/cellulose hydrogel exhibits a color transition upon sensing histamine (HI) vapor, along with a truth table of the logic analytical device for HI monitoring. Figure 5c features a photograph of a CNF@c-MOF double-layer supercapacitor device and an LED powered by devices in series under different deformations [135].

- Gene Therapy

Nonviral cationic materials, such as dendrimers, have been investigated for gene therapy to deliver genetic material into diseased cells. Dendrimers are nanosized synthetic polymers with numerous peripheral functional groups that can bind cationic moieties. They provide an alternative to viral carriers because of their biocompatibility and degradability in vivo [137,138].

- Biosensing

Two-dimensional cellulose-based materials have also demonstrated potential in biosensing applications. For example, heterostructures of these materials have been utilized in biosensing to leverage their distinctive electronic and surface properties for detecting a range of biological substances. Additionally, thin films of 2D materials have been suggested for use in surface plasmon resonance sensors to detect concentrations of toxic gases such as NO2, highlighting the adaptability of these materials in sensing applications [139,140].

- Case Studies and Real-World Examples

Cellulose-based 2D materials have real-world applications in the development of biosensors for environmental monitoring and healthcare diagnostics. For instance, cellulose nanocrystals and nanofibrils are being explored in hydrogels for biomedical uses like tissue engineering and regenerative biomedicine [100]. 

Two-dimensional materials have been utilized in cancer treatment for combination therapy, making use of their high drug-loading capacity and photothermal properties. These materials can be applied in multimodal therapy, encompassing drug delivery, photothermal therapy (PTT), and gene delivery, to enhance the effectiveness of cancer treatments [141,142]. 

**Figure 6:**
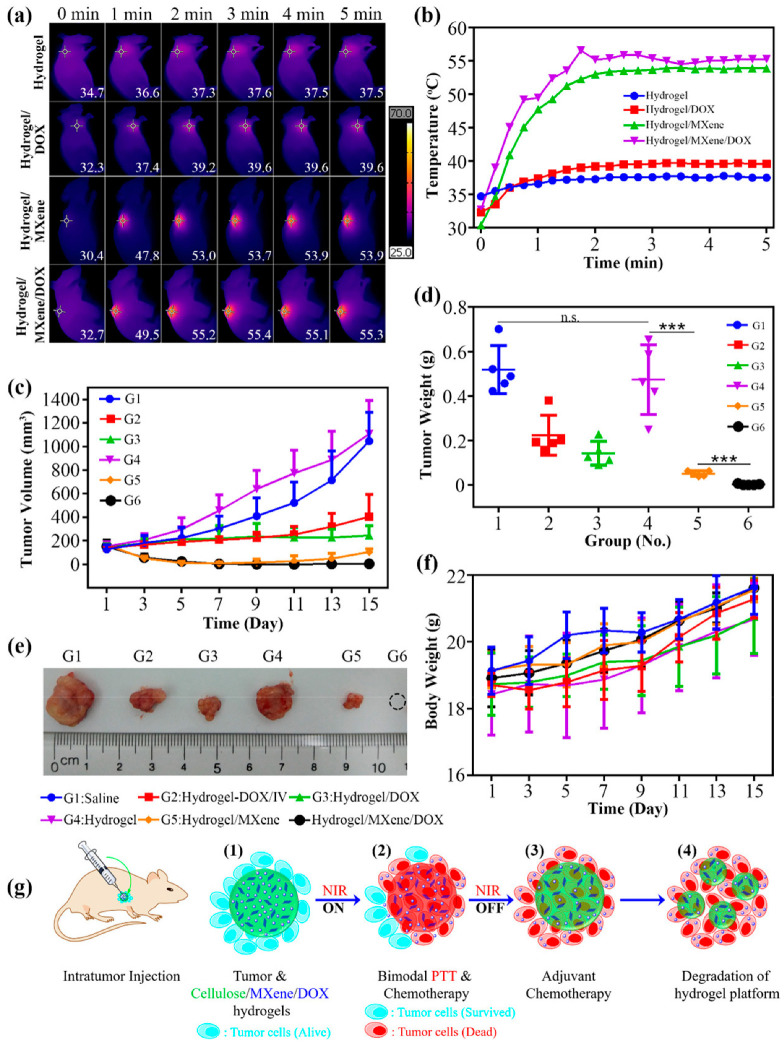
In vivo photothermal cancer therapy. (**a**) Graphic depiction of laser illumination and temperature at 0, 1, 2, 3, 4, and 5 min. (**b**) Temperature augmentation during laser illumination. (**c**) Tumor volumes during the observation period. (**d**) Tumor weights measured at day 15. Unpaired *t* test, *n* = 5, *** *p* < 0.001, ns stands for nonsignificant. Data are mean ± SD. (**e**) Representative images of tumors for each group. (**f**) Body weights of mice during different therapies. (**g**) Graphical representation of PTT/chemotherapy therapy. Intratumoral injection of cellulose/MXene/DOX hydrogels into mice followed by four steps: (1) cellulose/MXene/DOX hydrogels injected into the tumor site; (2) NIR irradiation on (PTT and chemotherapy); (3) NIR irradiation off (adjuvant chemotherapy); (4) degradation of the cellulose hydrogel platform [141]. Copyright with permission [Two-Dimensional MXene (Ti3C2)-Integrated Cellulose Hydrogels: Toward Smart Three-Dimensional Network Nanoplatforms Exhibiting Light-Induced Swelling and Bimodal Photothermal/Chemotherapy Anticancer Activity|ACS Applied Materials & Interfaces].

When ground into homogeneous slurries, cellulose-based hydrogels could be readily injected in vivo at the tumor sites in mice. Illumination (1.0 W/cm^2^, 5 min) of hepatocellular carcinomas was performed only twice (at day 1 and day 3) during a 2-week treatment period after the injection. The injected cellulose-based hydrogels included were DOX-loaded cellulose hydrogel (group 3, G3), neat cellulose hydrogel (group 4, G4), MXene-integrated cellulose hydrogel (group 5, G5), and DOX/MXene-containing cellulose hydrogel (group 6, G6). The MXene loading in G5 and G6 was fixed at 235.2 ppm. Two control groups were also investigated: saline (group 1, G1) and neat cellulose hydrogel combined with DOX (group 2, G2). Saline was administered by intravenous injection (G1). In G2, the neat cellulose hydrogel was intratumorally injected into mice, whereas the DOX solution was administered by intravenous injection. The anticancer activity of the hydrogels is further shown in Figure 6.

Figure 6a,b show that significant temperature increases were only detected for the hydrogels with incorporated MXene nanosheets (i.e., G5 and G6), demonstrating the outstanding photothermal properties of the MXene-integrated cellulose hydrogels in vivo. The FLIT values of cellulose/MXene hydrogel (G5) and DOX-loaded cellulose/MXene hydrogel (G6) reached temperatures in the range of 50–55 °C (Figure 6b), comparable to those obtained in Figure 6d for cellulose/MXene hydrogel with an MXene loading of 235.2 ppm. However, there were differences in both the temperature rise rate and plateau obtained in vivo (Figure 6b) compared with those measured in a cuvette (Figure 6d), possibly due to the different measurement methods employed. Figure 6c,d show the tumor growth curves and final tumor weights for each group. Figure 6e shows a representative tumor for all of the groups, and Figure 6f shows the body weight changes of the mice over the course of treatment. Notably, all six treatments had no significant influence on the body weight of mice.

A comprehensive analysis of Figure 6c–e confirms the effectiveness of the dual-modular cancer therapy. The similar tumor growth curves of group 1 (saline) and group 4 (hydrogel with NIR) imply that both saline and neat cellulose hydrogel were unable to inhibit tumor growth. The tumors in both group 2 (hydrogel combined with IV DOX) and group 3 (hydrogel/DOX) grew more slowly than the negative control (i.e., group 1 and group 4), indicating that both intravenously injected DOX and DOX released from the cellulose hydrogel can partially inhibit tumor growth but cannot effectively cure it. Similarly, comparing group 5 with group 4 showed that a single PTT can eliminate the vast majority of the tumor. Furthermore, the smaller tumors in group 5 compared to groups 2 and 3 suggested that PTT was more effective than DOX chemotherapy. However, tumors still relapsed after potent PTT (group 5 results). These results demonstrate that neither PTT nor chemotherapy alone was able to completely ablate the tumors and clear tumor cells. In contrast, tumors in group 6 were completely cleared and did not relapse, indicating that all the tumor cells were eradicated by the dual-modular PTT/chemotherapy. The noticeable relapse in group 5 but not in group 6 indicates that PTT could kill the majority of the tumor cells, but a small percentage of them may have survived (Figure 6g(2)), leading to tumor relapse, whereas the combined chemotherapy by DOX released from the hydrogel provided an adjuvant killing effect and a continuous inhibitory effect on the remaining tumor cells, achieving complete tumor clearance (Figure 6g(3)). Furthermore, as NIR illumination significantly accelerated the release rate of DOX in cellulose/MXene, more DOX may have been released into the tumor microenvironment during PTT, augmenting the effect of chemotherapy, resulting in a synergistic effect. Additionally, this cellulose-based platform can be partially disintegrated/degraded within 2 weeks, as shown in Figure 6g(4) [141].

In conclusion, cellulose-based 2D materials have a wide range of applications in the biomedical field due to their sustainable nature, biocompatibility, and versatile properties. Ongoing research is actively focused on developing these materials for drug delivery, gene therapy, and biosensing, among other applications, to overcome challenges and enhance their performance in real-world scenarios. 

### 9.2. Challenges and Limitations of Cellulose-Based 2D Materials 

Cellulose-based 2D materials have promising applications in various fields, but their widespread use is hindered by several existing challenges and limitations that will be discussed below.

- Scientific and Technological Challenges

One of the main scientific challenges involves the interaction of cellulose nanofibrils with bacteria and proteins, which leads to surface fouling. This interaction can cause the material to lose its integrity due to water-induced swelling [55,143]. Technological challenges include the high reflectivity of sunlight and low efficiency of photothermal conversion, which can significantly hinder the application of cellulose-based materials in solar water evaporation [144].

- Economic and Scalability Challenges

Economic and scalability challenges are prevalent in cellulose-based 2D materials production. The cost of production is high now, and scaling up while maintaining material quality is a big challenge. Additionally, these materials are currently limited to fillers in epoxy resins and polyurethane-based coatings, narrowing their potential market. However, there is hope that as applications broaden, the demand will increase, potentially reducing costs and expanding market opportunities [1,2].

- Potential Solutions and Ongoing Research

Ongoing research is currently focused on various strategies to overcome these challenges. For example, hybridization strategies are being explored to address the limitations of cellulose nanofibrils by combining multiple components that work together synergistically toward specific properties and applications [145]. 

Research is ongoing to enhance the efficiency of photothermal conversion by designing and optimizing the hierarchical structure of films through the addition of carbon materials during bacterial cellulose culture [133]. 

Research is currently focused on developing new methods for producing 2D materials such as graphene from sustainable microcrystalline cellulose at a low cost to address economic and scalability challenges [146]. Furthermore, active research in the exploration of MXenes as nanofillers in polymer-based coatings is ongoing [147]. 

In conclusion, although cellulose-based 2D materials show great promise for various applications, there are still significant challenges that need to be addressed. Nevertheless, ongoing research focused on overcoming these hurdles is paving the way for wider adoption of these materials in the future. 

### 9.3. Emerging Trends and Future Directions in Cellulose-Based 2D Materials

- Integration with Smart Drug Delivery Systems

Cellulose-based 2D materials are increasingly being integrated with smart drug delivery systems due to their adaptable surface chemistry, high surface area, biocompatibility, and biodegradability. These nanocellulose-based composite materials can be transformed into drug delivery carriers and designed into multidimensional structures such as 1D (nanofibers, microparticles), 2D (films), and 3D (hydrogels, aerogels) materials for use as different drug carriers. The specific requirements of these materials for drug delivery include good drug-loading capacity, biocompatibility, and biodegradability to ensure that drugs are released at the correct concentrations and proper rate [148].

- 3D Bioprinting

An emerging trend is the use of cellulose-based 2D materials in 3D bioprinting. A bioink that combines the exceptional shear-thinning properties of nanofibrillated cellulose with the rapid cross-linking ability of alginate has been developed for printing living soft tissue with cells in 3D. This advancement has allowed for the printing of both 2D grid-like structures and complex 3D constructs, including anatomically shaped cartilage structures such as a human ear and sheep meniscus using MRI and CT images as references. The potential application of nanocellulose in the 3D bioprinting of living tissues and organs has been convincingly demonstrated [149].

Table 7 provides a broad overview of the diverse applications of cellulose-based 2D materials across various sectors. It outlines how these sustainable, biodegradable materials are being leveraged in innovative ways, from environmental science to biomedical engineering and beyond. Each entry highlights specific applications, demonstrating the material’s versatility and adaptability, as well as the growing interest in exploiting its unique properties for cutting-edge technologies and solutions.

## 10. Real-World Applications and Implications

This section highlights the practical applications of hybrid cellulose-based films and addresses key questions regarding their commercial and regulatory status.

### 10.1. Commercialization and Regulatory Approval

- FDA Approval: To date, several cellulose-based products have been approved by regulatory agencies such as the FDA for various applications, including wound dressings and food packaging. However, specific hybrid cellulose-based materials are still undergoing extensive testing and evaluation to meet safety and efficacy standards for medical and pharmaceutical applications [150].
- Market Availability: Some cellulose-based materials, such as cellulose acetate and carboxymethyl cellulose, are commercially available and used in various industries. The development of hybrid cellulose-based materials with enhanced properties is an active area of research, with potential future commercialization [150].

### 10.2. Liability and Environmental Concerns

- Liability Issues: The liability for unintended consequences arising from the release of hybrid cellulose-based materials depends on regulatory compliance, product safety assessments, and the legal framework governing environmental protection and public health [150].
- Environmental Impact: The biodegradability of cellulose-based materials generally contributes to their environmental friendliness. However, the integration of non-biodegradable nanomaterials in hybrid films may raise concerns about their impact on ecosystems. Ongoing research aims to assess and mitigate potential environmental risks associated with these materials [150].

### 10.3. Case Study Highlights

- Biomedical Applications: Cellulose-based hydrogels and scaffolds have been successfully used in tissue engineering and wound healing, with ongoing research focused on enhancing their drug delivery capabilities [150].
- Environmental Remediation: Cellulose-based materials have been employed in water purification systems for the removal of pollutants, demonstrating their potential for addressing environmental challenges [150].

### 10.4. Future Research Opportunities and Potential Breakthrough Areas

Future research opportunities and potential breakthrough areas in the field of cellulose-based 2D materials include the following. 

Photomedicine for Cancer Treatment: Two-dimensional materials like graphene, boron nitride, and molybdenum disulfide have demonstrated great potential for photothermal therapy in cancer treatment. Their high surface area-to-volume ratio, biocompatibility, stability in physiological media, ease of synthesis and functionalization, along with high photothermal conversion efficiency make 2D nanomaterials excellent candidates for PTT [151]. 

Quantum Dots Based on 2D Materials: Gate-controlled quantum dot architectures are utilized in 2D materials and their heterostructures, offering the potential for electrical confinement, control, and manipulation of individual carriers within these materials. This not only enhances our understanding of spin-valley physics but also creates an optimal platform for exploring a wide range of condensed matter physics phenomena and realizing quantum computation in the 2D limit [56,152,153]. 

Protein and Carbohydrate-Based Materials for Anticancer Drug Delivery: New emerging trends in drug delivery design constantly report the use of carbohydrates such as cellulose and proteins in the recent literature. Additionally, drug vehicles combining carbohydrates and proteins have proven to be extremely effective, potentially impacting future therapeutic methods for curing cancer [135,154]. 

3D Bioprinting with Cellulose-Based Biomaterials Ink: Biomaterial ink derived from cellulose and used in 3D bioprinting has diverse applications, including tissue engineering, drug delivery, protein study, and the immobilization of microalgae, bacteria, and cells [155,156,157]. 

### 10.5. Environmental Impact and Sustainability of Cellulose-Based 2D Materials

- Environmental Implications

Cellulose-based 2D materials are sourced from renewable resources, offering an eco-friendly alternative to non-renewable materials. Cellulose is abundant, low-cost, biodegradable, and CO_2_ neutral, with a wide variety of fibers available globally. The production of these materials often involves bio-derived pathways developed with the environmental impact in mind. For example, the fabrication of a 2D TiO_2_/g-C_3_N_4_ heterojunction for generating 5-hydroxymethylfurfural (5-HMF) utilizes bio-derived pathways to access ionic liquids from natural renewable resources [158,159,160].

- Lifecycle Analysis and Recycling Potential

Cellulose-based 2D materials have an eco-friendly lifecycle because they are biodegradable. They can be recycled and reused in various applications, such as CO_2_ adsorption, where they exhibit high adsorption capacity. Following the adsorption process, the CO_2_ molecules can be desorbed, captured, and stored for further applications, showcasing the recycling potential of these materials [161].

- Eco-Friendly Production Methods

The production methods of cellulose-based 2D materials are often designed to be eco-friendly. For instance, producing 5-HMF from cellulose and glucose using a TiO_2_/g-C_3_N_4_/SO_3_H(IL) catalyst involves a minimum number of synthetic steps and uses readily available primary ingredients. The process is carried out under blue light-emitting diode (LED) radiation, which is energy-efficient [161,162]. 

Moreover, the production of cellulose-based thermal insulation materials involves recycling cellulosic and/or animal waste. These materials have demonstrated good thermal insulating quality, which can contribute to energy efficiency in buildings. In conclusion, cellulose-based 2D materials offer a positive environmental impact because of their renewable nature, biodegradability, and the eco-friendly methods used in their production. Their lifecycle includes the potential for recycling, further enhancing their sustainability. However, more research is needed to fully understand their environmental implications and to further improve their sustainability [2,163]. 

## 11. Concluding Statements

This review has explored the multifaceted advancements in hybrid cellulose-based films, highlighting their innovative applications in 2D nano-delivery systems. Although these materials present a promising avenue for applications across pharmaceuticals, environmental remediation, and biomedical engineering, it is important to acknowledge the challenges and limitations that accompany their development and use.

- Commercialization and Regulatory Considerations:

As of now, the journey towards widespread commercialization and regulatory approval, including FDA approval for medical applications, is ongoing. The potential for hybrid cellulose-based materials in the medical field is substantial, yet their adoption is contingent upon rigorous testing, validation, and regulatory compliance.

- Market Availability and Environmental Concerns:

The development of these materials into commercially available products is an active area of research. Efforts are being made to ensure that these innovative materials not only meet high performance standards but also address environmental sustainability and safety concerns.

- Addressing Liability and Safety:

Questions regarding liability and safety, particularly in the context of unintended environmental release, highlight the necessity for comprehensive risk assessments and the development of mitigation strategies to safeguard against potential adverse impacts.

Reflecting on these points, it becomes clear that the path forward for hybrid cellulose-based films involves not only scientific and technological innovation but also a concerted effort to navigate the complex landscape of regulatory, environmental, and ethical considerations. The optimism surrounding these materials must be tempered with a commitment to responsible research and development practices.

In summary, though the prospects for hybrid cellulose-based films are indeed promising, their successful integration into real-world applications requires a balanced approach that addresses both their potential benefits and the challenges they pose. Ongoing research and collaboration across disciplines will be crucial in realizing the full potential of these materials while ensuring their safe and sustainable use.

## Figures and Tables

**Table 1:** Key insights on cellulose-based 2D materials: properties, applications, and perspectives.

Aspect	Details
Material Base	Cellulose, recognized for its biocompatibility, versatility, and renewability.
Advancements	Integration with nanomaterials like nanoparticles, nanotubes, graphene, and advanced synthesis techniques.
Applications	Drug delivery, tissue engineering, environmental sustainability, and smart drug delivery systems.
Challenges	Scalability, compatibility with a broad range of compounds, regulatory hurdles, and environmental impact assessment.
Future Directions	Development of stimuli-responsive materials, integration with IoT for healthcare, and exploration of new synthesis methods to enhance properties.
Comparative Analysis	Relative to graphene and MXenes, cellulose-based 2D materials offer unique advantages in sustainability, biodegradability, and potential for diversification in applications.
Real-World Applications	Pharmaceutical industry, environmental remediation, biomedical scaffolds, energy storage, and water purification.
Regulatory and Commercialization Considerations	FDA approval process, market availability, liability issues, and environmental concerns.

**Table 2:** Comprehensive overview of cellulose film enhancement through nanomaterial integration.

Nanomaterial Type	Enhanced Properties	Applications	Outcomes	Key References
Nanoparticles	Optical properties, semiconductor effect	Smart packaging, optical sensors	Improved product shelf life, enhanced detection capabilities	[19,20]
Nanotubes (HNTs)	Mechanical properties, thermal stability	Reinforced composites, fire-resistant materials	Enhanced structural integrity, improved safety	[21,22]
Graphene (CNFs-FLG)	Young’s modulus, tensile strength, electrical conductivity	Electronic devices, conductive films	Increased device durability, better electrical performance	[23,24]
Manganese ferrite nanoparticles (MnFe_2_O_4_)	Piezoelectric sensitivity	Low-cost piezoelectric sensors	Enhanced sensitivity, potential for energy harvesting	[25,26]

**Table 3:** Enhancement strategies for cellulose films via surface modification.

Surface Modification Technique	Description	Outcomes	References
Grafting of Polymers	Utilization of starch acetate for creating controlled-release microcapsules containing gliclazide using an emulsion solvent evaporation technique.	Sustained drug release up to 24 h, dependent on microcapsule size, coat thickness, and SA concentration.	[29,30]
Coating with Bioactive Sub-stances	Use of biocompatible polymers (e.g., polylac-tic-co-glycolic acid, poly(ε-caprolactone), polylactic acid, poly(3-hydroxybutyrate-co-3-hydroxyvalerate), chitosan, cellulose for biomedical applications.	Improved biocompatibility, non-toxicity, reduced immunological reactions and side effects.	[31,32,33,34,35,36]
Other Chemical Modifications	Application of chitosan nanocomposites in drug delivery, tissue regeneration, and wound healing.	Enhanced biocompatibility and functionality.	[37]

**Table 4:** Advancements in and applications of cellulose-based systems in targeted delivery and antibacterial solutions.

Research Area	Key Findings	References
Customization of Cellulose Nanostructures	Potential for targeted drug delivery through one-dimensional nanostructures like nanorods, nanofibers, or nanotubes, with high drug-loading capacities and improved targeting.	[38]
Cellulose-Based Systems for Hydrophobic Drug Delivery	Development of antibacterial nanocrystalline cellulose through novel functionalization, enhancing antibacterial activity.	[39]
Antibacterial and Biofilm-Disrupting Hydrogels	Creation of highly absorbent antibacterial and bio-film-disrupting hydrogels for wound-dressing applications.	[40]
Antibacterial Pickering Emulsions	Development of antibacterial Pickering emulsions stabilized by bifunctional hairy nanocellulose.	[41]
Green Extraction of Antibacterial Cellulose-Based Nanofibers	Significant advancement in the eco-friendly extraction of antibacterial cellulose-based nanofibers from natural sources.	[42,43]
Superhydrophobic and Antibacterial Cellulose-Based Fibers and Fabrics	Progress in developing superhydrophobic and antibacterial cellulose-based fibers and fabrics for various applications.	[44]
Edible Carboxymethyl Cellulose Films with Antibacterial Agents	Edible carboxymethyl cellulose films incorporated with natural antibacterial agents for food-packaging applications.	[45]
Cellulose-Based Hydrogels for Antibacterial Wound Dressing	Identification of cellulose-based hydrogels as promising materials for antibacterial wound dressing.	[46]
Comparative Analysis with Other Nanomaterials	Comparative analysis highlights cellulose-based 2D materials’ unique advantages in sustainability, biodegradability, and potential for diversification in applications.	[46,47]

**Table 5:** Innovative cellulose-based materials for advanced applications.

Study Focus	Key Achievements	Methodology	Potential Applications	References
Customization of Cellulose Nanostructures	Tailoring for enhanced drug delivery via one-dimensional nanostructures.	Utilizing high surface-to-volume ratios of nanorods, nanofibers, or nanotubes for drug loading.	Targeted drug delivery systems.	[38]
Antibacterial Cellulose-Based Materials	Novel functionalization of nanocrystalline cellulose for prolonged antibacterial activity.	Integration of aldehyde groups into cellulose, enabling attachment of antibacterial agents.	Antibacterial materials and coatings.	[39]
Biofilm-Disrupting Hydrogels	Development of hydrogels with significant bactericidal efficacy for wound dressing.	Covalent bonding of ε-poly-L-lysine to cellulose hydrogels.	Wound-dressing materials with antibacterial properties.	[40]
Antibacterial Pickering Emulsions	Creation of emulsions stabilized by hairy nanocellulose for enhanced antibacterial properties.	Use of cellulose nanocrystals modified with aldehyde and carboxylic acid groups.	Antibacterial emulsions for healthcare and food safety.	[41]
Green Extraction of Antibacterial Nanofibers	Eco-friendly method to extract antibacterial cellulose-based nanofibers.	Thermo-oxidation with H_2_O_2_ to extract nanofibers from pine cones.	Green production of antibacterial fibers for medical and environmental applications.	[42,43]
Superhydrophobic and Antibacterial Fibers	Enhancing cellulose-based fibers and fabrics with superhydrophobic and antibacterial properties.	Surface structuring and chemical modifications to introduce antibacterial agents.	Textiles with antibacterial and water-resistant features.	[44]
Edible Carboxymethyl Cellulose Films	Development of edible films with natural antibacterial agents for food packaging.	Incorporation of lysozyme into carboxymethyl cellulose films.	Edible food packaging with antimicrobial properties.	[45]
Cellulose-Based Hydrogels for Wound Dressing	Identifying hydrogels as promising materials for antibacterial wound dressing.	Developing cellulose-based hydrogels mimicking the skin’s microenvironment.	Biocompatible wound-dressing solutions.	[46]
Hollow Mesoporous Silica Particles	Introducing porous silica materials for drug delivery and dye removal.	Bio-template-directed method with cellulose nanocrystals as the template.	Therapeutic nanoparticles and environmental purification.	[47]
Stimuli-responsive Chitosan Architectures	Exploring chitosan’s adaptability for controlled drug delivery via stimulus responsiveness.	Designing chitosan-based architectures for bioactive agent delivery under specific conditions.	Smart drug delivery systems responding to physiological changes.	[48]
Hairy Nanocellulose Supramolecular Structures	Creation of structures for sustained drug molecule release with pH responsiveness.	Cross-linking beta-cyclodextrin ethylene diamine with hairy nanocellulose.	pH-responsive drug delivery platforms.	[49]
Internally Bridged Nanosilica for Drug Hosting	Developing colloids with distinctive structures for drug delivery.	Utilizing monodisperse colloids with a sea urchin-like structure for internal drug hosting.	Novel nanocarriers for controlled drug release.	[50]
Hollow Silica-Based Drug Delivery Optimization	Investigating drug transfer processes using hollow silica particles.	Molecular dynamics simulations to enhance drug delivery efficiency.	Optimized drug delivery vehicles for therapeutic applications.	[51]
Chitosan/Hyaluronic Acid Nanoparticles	Developing nanoparticles for the encapsulation and controlled release of molecules.	Oxidative stress and pH-responsive design for targeted therapeutic applications.	Targeted nanoparticle systems for drug and protein delivery.	[52]
Reusable Green Adsorbent Aerogel	Eco-friendly adsorbent aerogel for dye removal, highlighting environmental applications.	Crosslinking hairy nanocrystalline cellulose with modified chitosan.	Sustainable materials for water purification and environmental remediation.	[53]
Stimuli-responsive Chitosan for Bioactive Agents	Reviewing the potential of stimuli-responsive chitosan as an efficient drug delivery platform.	Designing chitosan architectures for targeted bioactive agent release.	Controlled and targeted delivery of bioactive agents in biomedical applications.	[54]

**Table 6:** Comparative analysis of cellulose-based 2D materials and other nanomaterials.

Property	Cellulose-Based 2D Materials	Other Nanomaterials	References
Abundance and Cost	High; low-cost due to abundant renewable sources	Low; relatively higher cost and less abundant	[74]
Environmental Friendliness	Highly biodegradable and environmentally friendly	Less biodegradable, varying environmental impact	[78,81]
Electrical Conductivity	Generally low but can be modified for specific applications	High; especially in graphene for electronics	[79]
Thermal Stability	Suitable for packaging and insulation, though less than CNTs	Exceptional in CNTs for high-temperature applications	[80]
Biodegradability	Superior; advantageous for eco-friendly applications	Generally lower; less suitable for such applications	[81]
Applications	CO_2_ adsorption, supercapacitors, biomedical, etc.	Biomedical, electronic applications, etc.	[75,78]

**Table 7:** Comprehensive overview of cellulose-based 2D materials applications.

Sector	Application	Description	References
Environmental Science	CO_2_ adsorption	Utilizing cellulose-based 2D materials for capturing and storing carbon dioxide due to their high adsorption capacity.	[75]
Energy Storage	Supercapacitors	Creating composite electrodes with MXenes for enhanced energy storage capabilities.	[75]
Biomedical Engineering	Drug delivery systems	Developing smart drug delivery systems using cellulose-based materials for controlled drug release.	[56,148]
3D Bioprinting	Printing living tissues and organs	Using cellulose-based bio-inks for 3D bioprinting complex structures, including tissues and organs.	[149]
Pharmaceutical	Smart drug delivery	Integrating cellulose materials with drug delivery systems for efficient, targeted therapies.	[148]
Water Purification	Filtration technologies	Applying cellulose-based materials in water purification systems for removing contaminants.	[75]
Innovative Technologies	4D printing	Exploiting the properties of cellulose-based materials in 4D printing for dynamic, responsive structures.	[115,116]

## Data Availability

Not applicable.

## List of Symbols and Abbreviations

2D	Two-dimensional
CNC	Cellulose nanocrystals
CNF	Cellulose nanofibers
CNT	Carbon nanotubes
FLG	Few-layer graphene
HNC	Hairy nanocrystalline cellulose
HNT	Halloysite nanotubes
LED	Light-emitting diode
LCA	Life cycle analysis
MXene	A class of 2D transition metal carbides, nitrides, or carbonitrides
PVP	Polyvinylpyrrolidone
SA	Starch acetate
SNEDDS	Self-nanoemulsifying drug delivery system
UV	Ultraviolet
SEM	Scanning Electron Microscopy
TEM	Transmission Electron Microscopy
FTIR	Fourier-Transform Infrared Spectroscopy
